# Preparation of a Vertical Graphene-Based Pressure Sensor Using PECVD at a Low Temperature

**DOI:** 10.3390/mi13050681

**Published:** 2022-04-27

**Authors:** Xin Cao, Kunpeng Zhang, Guang Feng, Quan Wang, Peihong Fu, Fengping Li

**Affiliations:** 1 College of Mechanical and Electrical Engineering, Wenzhou University, Wenzhou 325035, China; 194511481355@stu.wzu.edu.cn (X.C.); iamfph@163.com (P.F.); 2 Zhejiang Provincial Engineering Center of Laser and Optoelectronic Intelligent Manufacturing, Wenzhou University, Wenzhou 325035, China; zkp@wzu.edu.cn (K.Z.); fengguang0150@link.tyut.edu.cn (G.F.); 3 College of Mechanical Engineering, Jiangsu University, Zhenjiang 212013, China; wangq@mail.ujs.edu.cn

**Keywords:** flexible, pressure, vertical graphene (VG), sensor

## Abstract

Flexible pressure sensors have received much attention due to their widespread potential applications in electronic skins, health monitoring, and human–machine interfaces. Graphene and its derivatives hold great promise for two-dimensional sensing materials, owing to their superior properties, such as atomically thin, transparent, and flexible structure. The high performance of most graphene-based pressure piezoresistive sensors relies excessively on the preparation of complex, post-growth transfer processes. However, the majority of dielectric substrates cannot hold in high temperatures, which can induce contamination and structural defects. Herein, a credibility strategy is reported for directly growing high-quality vertical graphene (VG) on a flexible and stretchable mica paper dielectric substrate with individual interdigital electrodes in plasma-enhanced chemical vapor deposition (PECVD), which assists in inducing electric field, resulting in a flexible, touchable pressure sensor with low power consumption and portability. Benefitting from its vertically directed graphene microstructure, the graphene-based sensor shows superior properties of high sensitivity (4.84 KPa^−1^) and a maximum pressure range of 120 KPa, as well as strong stability (5000 cycles), which makes it possible to detect small pulse pressure and provide options for preparation of pressure sensors in the future.

## 1. Introduction

With an increasing number of wearable devices being used in heart rate monitoring, industrial robots, and medical electronic monitoring, flexible sensors with superior performance have developed rapidly [1,2,3,4]. Generally speaking, the working mechanisms of common pressure sensors include the piezoresistive effect, capacitance effect, and resistance effect [5,6,7,8]. Among them, the key to realizing pressure sensing by using the piezoresistive effect is that the external load will cause certain deformation of the microstructure of force-sensitive material, which results in changes in the carrier mobility to achieve a change in the actual resistance [9]. This usually occurs by applying a fixed or scanning voltage under different pressures to obtain a corresponding relationship between pressure and current. For the shortcomings of a bloated volume and serious linear drift in the internal structure of traditional pressure sensors, a large number of researchers have turned their attention to pressure sensors made of smaller and more responsive two-dimensional materials [10,11]. Additionally, wearable pressure sensors required experience comfortable and wear resistance that is directly fed back to the selection of packaging materials [12,13,14,15,16,17]. The wearable demand requires that the pressure sensor must use materials with superior performance in terms of features such as bending, good flexibility, fatigue resistance, etc. The packaging substrate typically includes polydimethylsiloxane (PDMS), polyethylene glycol terephthalate (PET), polyimide (PI), etc. [18,19,20,21]. In order to improve the performance of the sensor, the design can only be optimised in terms of sensing structure and material type; therefore, some two-dimensional lightweight materials that meet the requirements are constantly tried by researchers to apply to sensors. [22,23].

As a two-dimensional carbon material, graphene is often used as a force-sensitive material for pressure sensors because of its excellent mechanical and electrical properties [24]. The general methods for preparing graphene include chemical vapor deposition (CVD), liquid-phase exfoliation, and SiC epitaxial growth. Among them, liquid-phase exfoliated graphene usually has the disadvantages of structural defects and uncontrollable shape [25]. Although SiC epitaxial growth of graphene can produce high-quality graphene, it requires expensive substrate cost and harsh conditions of high temperature (1500 °C) and ultra vacuum, which is not conducive to mass production in the industry [26]. Compared with the previous two methods, the preparation of graphene in CVD is a more reliable method in terms of cost and material quality [27,28]. However, the growth of graphene by CVD still needs a metal substrate as a catalyst; otherwise, the growth temperature needs to be higher (1100~1650 °C) [29,30]. For example, the temperature required to grow graphene on the most commonly used copper substrate is about 1050 °C [31], which makes any known flexible packaging substrate unable to exist directly as a growth substrate. Therefore, graphene prepared generally by CVD as force-sensitive material must undergo the process of transferring from the growth substrate to the target substrate, which may cause certain damage to the internal structure and surface contamination of the material [32]. To solve this problem, a flexible substrate with high-temperature resistance can avoid the complex and unnecessary transfer process. In plasma-enhanced chemical vapor deposition (PECVD), the high-energy electrons produced by the plasma ionize, excite, and dissociate the carbon-containing precursor [33]. The free radicals produced by this dissociation reaction are more active than the free radicals cleaved at high temperatures in conventional CVD, so graphene can be prepared at relatively low temperatures without a catalytic substrate [34]. In addition, plasma generators are divided into three categories due to different power supply frequencies for generating plasma; radio frequency (RF) plasma has the advantage of high energy density and a larger plasma volume, thus yielding high growth rates [35].

Here, we propose an ultra-high-performance, piezoresistive sensor assembled by using PECVD growth of VG at a low temperature (650 °C) that has the characteristics of smooth texture and small volume. The sensor consists of mica paper, interdigital electrode, VG, PDMS, and copper wire. Among them, mica paper is an insulating flexible substrate for growing graphene without subsequent transfer. The interdigital electrode is used to ensure full contact with VG and external voltage, and PDMS is used to strictly flexible seal the whole device. The structure’s combined PDMS–VG–PDMS sandwich can successfully benefit from the resistance of high temperature and the bending flexibility of mica paper so that the sensor can achieve high sensitivity (4.84 KPa^−1^) and an ultrawide pressure-monitoring range (0~120 KPa). In addition, the sensor also has superior durability (5000 cycles). Further noteworthy is that its extremely low cost and tiny volume are conducive to mass production; thus, the method of directly using growth substrate as packaging material provides a direction for future sensors.

## 2. Materials and Methods

### 2.1. Fabrication of Vertical Graphene (VG) Layer

The VG was fabricated by PECVD with assisting electric field applied to propel the vertical growth of graphene. A piece of mica paper (purchased from Yangzhou Zhong Bo Metallurgical Science and Technology Co., Ltd., Yangzhou, China) substrate (thickness of 80 μm and width of 2 cm × 2 cm) was ultrasonically cleaned with deionized water for 10 min, then quickly blow-dried in nitrogen, and were placed in a dust-free magnetic suction box. A layer of the interdigital electrode with a thickness of 10 μm was electroplated on the center of the mica paper on one side and applied to a flexible insulated electrode substrate (FIES) that consists of 90% Cu, 8% Ag, and 2% Au. The FIES were placed on a plane Petri dish in the constant temperature chamber of PECVD with its electrode side faces up. Then, the tubular furnace chamber was evacuated to 1 × 10^−4^ Torr, Ar (200 sccm), and H_2_ (10 sccm) gases with an inflow ratio of 20:1 until the overall air pressure increases to atmospheric pressure. Next, the atmospheric pressure was maintained, and the flow ratio was specified while gradually heating the temperature of the chamber support dish to 650 °C. For pretreatment, the plasma was generated via the RF power, and a vertical electric field was added with a frequency of 13.5 MHz. 1 sccm carbon source gas of CH_4_, which penetrated the chamber for 5 h, to grow deposited graphene nanowalls. After the chamber cooled to room temperature, the RF power supply was turned off, followed by the electric field, CH_4_, H_2_, and Ar, in turn, thereby obtaining the grown VG layer.

### 2.2. Fabrication of Vertical Graphene-Polydimethylsiloxane (VG–PDMS) Flexible Sensor

The sensor is made of a flexible mica paper substrate with a double-layer PDMS packaging with a VG layer. First, 5 mL PDMS (Sylgard 184, DowCorning, Midland, MI, USA) and 500 μL PDMS curing agent (Sylgard 184, DowCorning, Midland, MI, USA) were mixed and stirred adequately in a flask until a large number of tiny bubbles appear; then, the mixture was placed in the sealing chamber, pumping to 10^−4^ Torr for removing bubbles. Afterward, the PDMS mixture was tiled in a prepared molding box with a thickness of 1 mm and cured at 60 °C for 3 h. Two copper wires (diameter of 0.5 mm and length of 15 cm) were fixed at both ends of the interdigital electrode on the substrate of mica paper by conductive silver pulp (purchased from Nanhai Qiming Guangda Technology Co., Ltd., Shenyang, China). The material layer of the external lead was placed onto the cured PDMS, and then the same proportion of PDMS mixture was deployed to defoam and poured into the shaping box until the liquid tiles were 1 mm above the mica substrate; the mixture continued to solidify for 3 h. Finally, the redundant PDMS layer was removed at the edge, and a flexible vertical graphene sensor (FVGS) was obtained, with a size of 2 cm × 2 cm.

### 2.3. Characterization

The crystalline structure of the prepared VG was characterized by field-emission scanning electron microscopy (FE-SEM, S4700, Hitachi, Tokyo, Japan) and a Raman spectrometer (LabRAM HR800, Horiba, France), equipped with an objective (50×) using a He-Ne laser (λ = 532 nm). The macroscopic images of the sample before and after growth were investigated by a Zeiss microscope (Axioscope 5, Zeiss, Oberkchen, Germany) under the 10× lens. The sheet resistance of the VG layer on the mica paper and the interdigital electrode was measured by using four-point-probe methods via a Keithley 2400 source meter. The circulatory tests of the electromechanical properties of the FVGS were carried out by taking Keithley 2400 source meters with two-point-probe equipment.

### 2.4. Performance Test of Flexible VG Pressure Sensor

The self-made system consists of a ZhiQu pressure tester (ZQ-21A-2, ZhiQu, DongGuan, China), a computer-controlled stepping motor (TD110A12-60A, Tuojiade technology, Shenzhen, China), and an accurate digital source meter (Keithley 2400, Tektronix, Beaverton, OR, USA) to measure the real-time response of FVGS at different pressures. The pressure applied to the sensor was measured by a computer-controlled stepping monitor to add a process to the pressure tester. The corresponding response current was measured by using a source meter (Keithley 2400, Tektronix, USA), and a computer-controlled stepping motor was used to evaluate mechanical stability.

## 3. Results and Discussion

**Figure 1:**
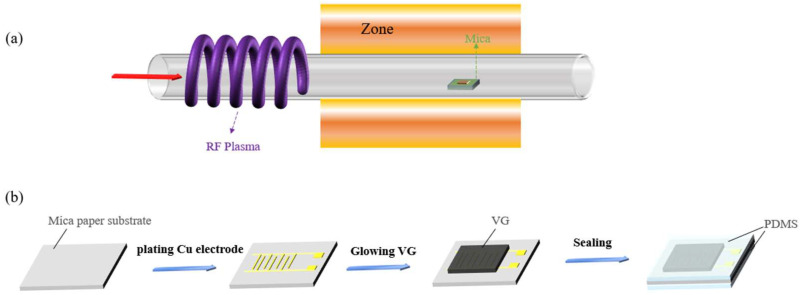
Preparation process of sensitive layer material and sensor: (**a**) schematic illustration of the growth of vertical graphene(VG) in plasma-enhanced chemical vapor deposition (PECVD); (**b**) fabrication procedure of the flexible vertical graphene-based (VG-based) pressure sensor.

Figure 1a represents the schematic diagram of the experimental device and growth process. First of all, the VG nanowall produced by PECVD depends on the type of plasma source in shape and structure. We chose an RF plasma generator with 250 W power as the plasma source, which can provide high energy density and a larger plasma volume. Owing to its low price, high purity, and fewer by-products than other carbon source gases, CH_4_ was chosen as the carbon source gas, which is conducive to the growth of VG. Ar and H_2_ were chosen as the protective gas and the reducing gas, respectively. As an inert gas, Ar protects the stability of the reaction in the chamber and maintains the pressure and molecular mixing ratio in the chamber. Before the addition of CH_4_, the chamber needs to be filled with Ar gas to exhaust the excess air in the reaction chamber, gradually raising the temperature to 650 °C (the temperature required for VG growth) within the next hour at atmospheric pressure. CH_4_ produces a large number of activated carbon groups under the bombardment of the plasma. With the movement of molecules, it gradually nucleates and migrates on the electrode mica substrates to maintain the continuous growth of VG. In the past, copper and other catalytic substrates were used as substrates for growing VG. In the process of transferring graphene from a catalytic substrate to a flexible substrate, it may cause some damage to the integrity of its structure. Moreover, the temperature of growing graphene directly on flexible substrates in a conventional CVD is about 1050 °C, which is not acceptable for ordinary flexible substrates. Therefore, we utilized PECVD to reduce the temperature of growing the required VG and a flexible mica paper substrate with superior resistance to a high temperature (700~800 °C) for a long time, which solve the problem of transfer and substrate resistance in an ultrahigh temperature.

The manufacturing process of the whole sensor part is shown in Figure 1b. As can be seen from left to right, the plating composition on standard size (2 cm × 2 cm) flexible mica paper was mainly copper interdigital electrode, and the substrate was put into the PECVD for growing expected VG. The growth of VG completely covered the interdigital electrode and mica paper. Conductive silver pulp was used to point copper wire (the length of 10 cm) at both ends of the interdigital electrode. The double-layer 1 mm thick PDMS was used finally to package. The whole process completed the flexible, wearable requirements without additional transfer materials; therefore, it is a valuable method to learn.

**Figure 2:**
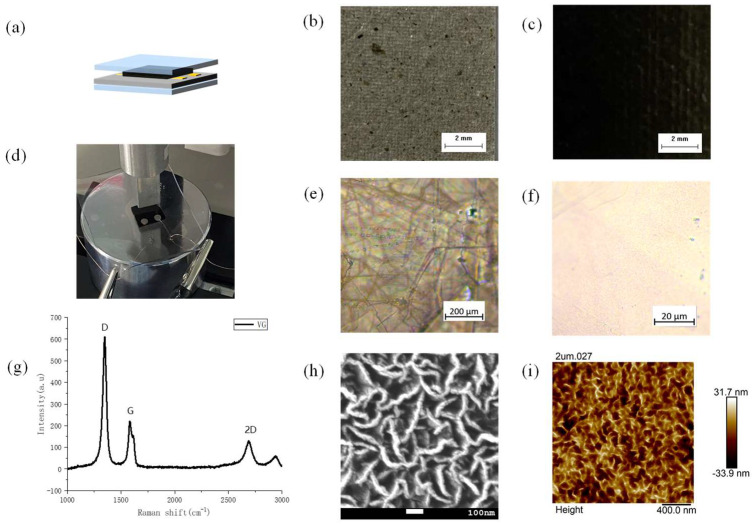
Device structure and material characterization: (**a**,**d**) structural diagrams and real images of the sensor, respectively; (**b**,**c**) images of before and after the growth of mica substrate VG; (**e**,**f**) images of VG under the light mirror; (**g**) the Raman spectrogram of VG; (**h**) the scanning electron microscopy (SEM) diagram of VG; (**i**) AFM diagrams of VG.

The schematic of FVGS is shown in Figure 2a. The overall thickness and size of the sensor device ensure sufficient wear resistance and flexibility because it reaches 2.3 mm with the support of double-layer PDMS. The actual image of the electrical performance test is shown in Figure 2d, and the total force area is 1 cm^2^. As a carrier base, the mica paper was mainly made of calcium carbonate dolomite and paper mortar after thermochemical stripping and high-temperature forging, followed by cooling, which is a process that has the advantages of good heat resistance (700~800 °C) and flexibility. Thus, it is a flexible substrate worth using as a growth VG. Figure 2b,c show the comparison before and after the end of growth on the mica paper. It shows that the material has almost covered the substrate. To verify that the growth material on the mica paper substrate is VG instead of other carbon-based derivative impurities, we used a Zeiss microscope to record images before growth (Figure 2e) and post-growth (Figure 2f), respectively. Figure 2g shows the results of the Raman spectroscopy. The VG causes a slightly strong G peak at 1580 cm^−1^ and a strong defect peak (D peak) at 1350 cm^−1^. The G peak is caused by vibration in the sp2 carbon atom surface, which can effectively reflect the number of layers of graphene. The 2D peak of about 2700 cm^−1^ proves that the material is graphene, which mainly indicates the interlayer stacking mode of carbon atoms. The structure on the top of the VG observed by SEM under the 5000-back magnification is shown in Figure 2h, achieving the expected vertical growth. Figure 2i is an AFM diagram analyzed with nanoscope software, and the height of the VG is 2 um. Since the growth direction of graphene is vertical, the edge of VG in the later stage is no longer as flat as the edge in the film with continuous growth. It can directly be observed from the AFM diagram that it presents a petal shape.

**Figure 3:**
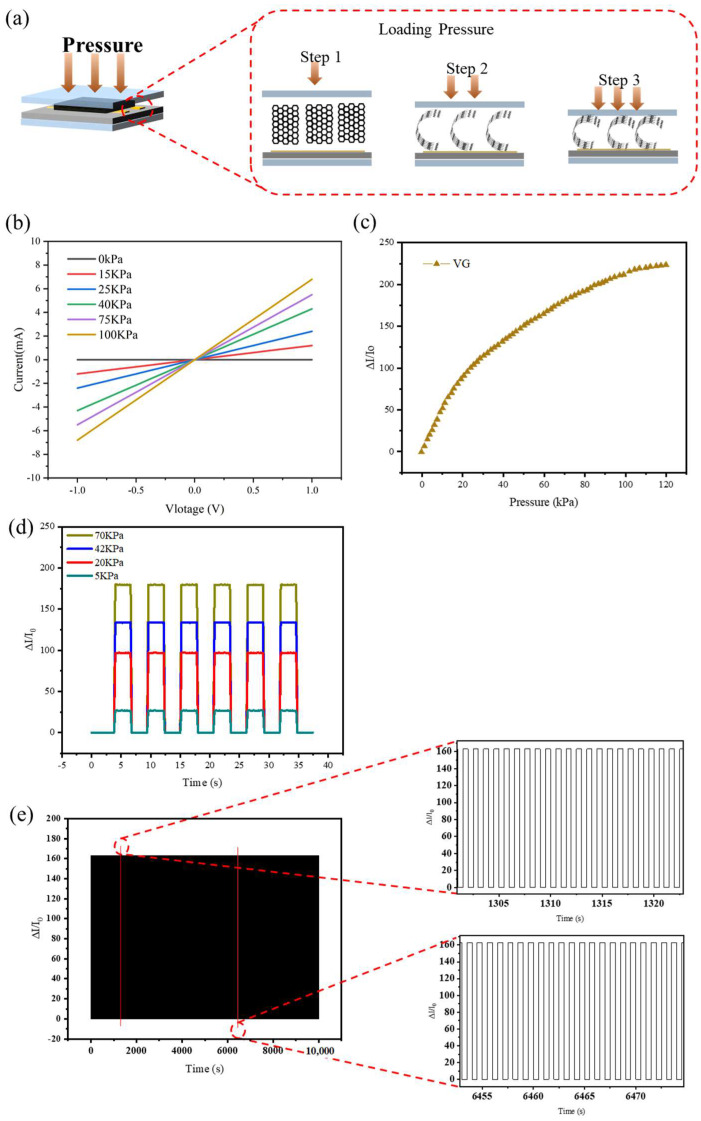
Sensing performances of the flexible VG sensor: (**a**) schematic diagram of the working mechanism of a flexible pressure sensor; (**b**) *I−V* curves of the sensor with applied pressure including 0 KPa, 15 KPa, 25 KPa, 75 KPa, and 100 KPa; (**c**) current response (∆*I/I*_0_) vs. applied pressure; (**d**) current changes in sensors at different pressures (5, 20, 42, 70 KPa); (**e**) cycling test under external pressure of 60 KPa for 5000 cycles.

The schematic diagram of the pressure sensing model structured with PDMS/VG-Mica/PDMS is shown in Figure 3a. The pressure causes the horizontal bending of VG nanowall, which changes the crystal layer spacing of graphene, leading to changes in carrier mobility. The sensor has a resistance value without loading pressure. Therefore, the relationship between current and pressure variables needs to be studied. The *I–V* curve can be obtained by increasing the voltage value from −1 V to 1 V under different constant load pressures (0 KPa, 15 KPa, 25 KPa, 40 KPa, 75 KPa, and 100 KPa). As shown in Figure 3b, the *I–V* curves of the sensor under different pressures show a relatively stable, linear relationship. With the increase in pressure, the resistance value (curve slope) increases, indicating that its good ohmic characteristics are independent of input voltage.

To better demonstrate the performance of the prepared sensor device, sensitivity *S* = (∆*I/I*_0_)/*δP* was introduced, where the ∆*I* is the current variable, the *I*_0_ is the initial current value at zero pressure, and the *δP* is the pressure variable. The changing trend of sensitivity is shown in Figure 3c. It can be seen that the sensitivity of the first half (0~20 KPa) can reach 4.84 Kpa^−1^, and the second half (>20 Kpa) belongs to the nonlinear region of sensitivity. High sensitivity is caused by the good recovery performance of graphene, coupled with good conduction of pressure by flexible PDMS. More notably, the stable and repeatable current response of the pressure sensor under pressure changing from 5 KPa to 70 KPa in Figure 3d further illustrates the reliability of the sensor. Figure 3e shows the cyclic performance of the prepared sensor. Under the pressure of 60 KPa, 5000 cycles were carried out, indicating that the manufactured sensor has good reliability and stability. From the perspective of overall performance, it can provide an option for the future field of flexible sensor materials.

Overall, the material cost required to prepare the sensor roughly includes mica paper, PDMS and curing agent, carbon source, and other gases. These are easy to obtain and relatively cheap, compared with other preparation methods. The packaged device can obtain considerable response under no more than 1 V. It can be seen that its power consumption is also very small, which provides the possibility for mass industrial production in the future. As for the sensitivity, compared with 0.19 KPa^−1^ of ZnO/double-layer doped graphene sensor [36] and 0.96 KPa^−1^ of V-shaped graphene sensor [37], the sensor can reach 4.84 KPa^−1^. In addition, the overall pressure detection range (~120 KPa) is also wider than those of the abovementioned sensors. The most important point is that the development direction of the flexible sensor in the future is to fit the skin, which makes the flexible growth substrate directly used as packaging material to realize the coordinated coexistence of VG structural integrity and flexibility. There may be more suitable growth substrates to achieve higher performance graphene sensors in the future, but this is also a bold attempt.

## 4. Conclusions

In summary, we demonstrated a piezoresistive sensor assembled by using the PECVD method, with low-temperature growth of VG, which has the characteristics of a smooth structure and small volume. It consists of mica paper, interdigital electrode, VG, PDMS, and copper wire. Mica paper is an insulating, flexible substrate for the growth of graphene, without a subsequent transfer. Interdigital electrodes were used to ensure full contact with VG and external voltage. PDMS was strictly and flexibly sealed to the whole device. The structure’s combined PDMS sandwich can successfully make use of the high-temperature resistance and bending flexibility of mica paper so that the sensor can achieve high sensitivity (4.84 KPa^−1^) and ultrawide pressure monitoring range (0~120 KPa). In addition, the sensor has superior durability (5000 cycles). Further noteworthy is that the sensor’s extremely low cost and tiny volume are conducive to its mass production. Therefore, the method of directly using growth substrate as packaging material provides a direction for future sensors.
